# Neural network-based arterial diameter estimation from ultrasound data

**DOI:** 10.1371/journal.pdig.0000659

**Published:** 2024-12-02

**Authors:** Zhuangzhuang Yu, Manolis Sifalakis, Borbála Hunyadi, Fabian Beutel

**Affiliations:** 1 Department of Signal Processing & Modelling, imec The Netherlands / Holst Centre, Eindhoven, The Netherlands; 2 Department of Microelectronics / Signal Processing Systems, Delft University of Technology, Delft, The Netherlands; 3 Department of Health Research, imec The Netherlands / Holst Centre, Eindhoven, The Netherlands; Almoosa College of Health Sciences, SAUDI ARABIA

## Abstract

Cardiovascular diseases are the leading cause of mortality and early assessment of carotid artery abnormalities with ultrasound is key for effective prevention. Obtaining the carotid diameter waveform is essential for hemodynamic parameter extraction. However, since it is not a trivial task to automate, compact computational models are needed to operate reliably in view of physiological variability. Modern machine learning (ML) techniques hold promise for fully automated carotid diameter extraction from ultrasonic data without requiring annotation by trained clinicians. Using a conventional digital signal processing (DSP) based approach as reference, our goal is to (a) build data-driven ML models to identify and track the carotid diameter, and (b) keep the computational complexity minimal for deployment in embedded systems. A ML pipeline is developed to estimate the carotid artery diameter from Hilbert-transformed ultrasound signals acquired at 500Hz sampling frequency. The proposed ML pipeline consists of 3 processing stages: two neural-network (NN) models and a smoothing filter. The first NN, a compact 3-layer convolutional NN (CNN), is a region-of-interest (ROI) detector confining the tracking to a reduced portion of the ultrasound signal. The second NN, an 8-layer (5 convolutional, 3 fully-connected) CNN, tracks the arterial diameter. It is followed by a smoothing filter for removing any superimposed artifacts. Data was acquired from 6 subjects (4 male, 2 female, 37 ± 7 years, baseline mean arterial pressure 86.3 ± 7.6 mmHg) at rest and with diameter variation induced by paced breathing and a hand grip intervention. The label reference is extracted from a fine-tuned DSP-based approach. After training, diameter waveforms are extracted and compared to the DSP reference. The predicted diameter waveform from the proposed NN-based pipeline has near perfect temporal alignment with the reference signal and does not suffer from drift. Specifically, we obtain a Pearson correlation coefficient of *r* = 0.87 between prediction and reference waveforms. The mean absolute deviation of the arterial diameter prediction was quantified as 0.077 mm, corresponding to a 1% error given an average carotid artery diameter of 7.5 mm in the study population. This work proposed and evaluated an ML neural network-based pipeline to track the carotid artery diameter from an ultrasound stream of A-mode frames. By contrast to current clinical practice, the proposed solution does not rely on specialist intervention (e.g. imaging markers) to track the arterial diameter. In contrast to conventional DSP-based counterpart solutions, the ML-based approach does not require handcrafted heuristics and manual fine-tuning to produce reliable estimates. Being trainable from small cohort data and reasonably fast, it is useful for quick deployment and easy to adjust accounting for demographic variability. Finally, its reliance on A-mode ultrasound frames renders the solution promising for miniaturization and deployment in on-line clinical and ambulatory monitoring.

## Author summary

The carotid artery diameter waveform is highly relevant for cardiovascular diagnostics and typically acquired using ultrasound imaging. Our work focuses on a novel machine learning-based approach to track the carotid artery diameter in ultrasound data. Opposed to conventional digital signal processing strategies, which require manual fine-tuning, a key advantage of the machine learning approach (implemented as a sequence of neural network models) is the automated learning process. Going even further, we combine the strength of automated learning with relevant domain knowledge on identifying the anatomy of interest, such that the devised models do not require a large dataset to learn from. Eventually, the evaluation of the proposed method results in merely a 1% deviation of the identified and tracked carotid diameter in comparison to reference data. Not only do we achieve an effective tracking performance, but we also foresee the models to be computationally affordable and embedded on small-size devices like wearables for application outside of the clinical settings.

## Introduction

### Background

Cardiovascular Diseases (CVDs) are the leading cause of premature human mortality globally [1]. CVDs are posing an insidious danger as they typically evolve asymptomatically before having lethal consequences like strokes and heart attacks, or leading to costly chronic conditions like kidney disease and neural impairments from brain damage [2]. Various interventions to assess CVD risk involve the assessment and monitoring of the carotid artery health by means of ultrasonography. Besides conventional hemodynamics like arterial blood flow (e.g., to detect stenosis), arterial stiffness based on pulse wave velocity measurements is becoming increasingly relevant as biomarker in CVD diagnostics and prevention as it reflects the vascular aging and individual susceptibility to hypertension-mediated organ damage [3,4]. However, in the first instance, these markers require high-quality arterial diameter waveforms, which subsequently feed into advanced analyses of the pulse waveform.

### Prior Work & State of the Art

Although the understanding of arterial physiology and hemodynamics is well developed, and advanced imaging modalities are available, novel methods towards fully automated tracking of the carotid artery and its distension are lacking in clinical practice. Given that arterial monitoring is typically accompanied by motion artifacts (e.g., resulting from breathing, swallowing, or coughing), precision tracking to obtain detailed information on arterial dynamics and mechanical properties like elasticity remains challenging. To this end, research has established ultrasound as a non-invasive, safe, yet reliable method for imaging of soft tissues like arteries, with the required resolution and accuracy for precision measurements also over longer periods of time [5–7]. However, current clinical use of ultrasound imaging relies on bulky infrastructure and premises a skilled operator with anatomical domain knowledge to manually annotate the arterial wall positions from the images. This manual dependency makes the process costly, time-consuming, thus unscalable to large widespread screening or long-term monitoring. To overcome these limitations, recent research based on digital signal processing (DSP) algorithms has strived to enable (semi-)automatic tracking of the carotid artery diameter [8–18].

**Fig 1:**
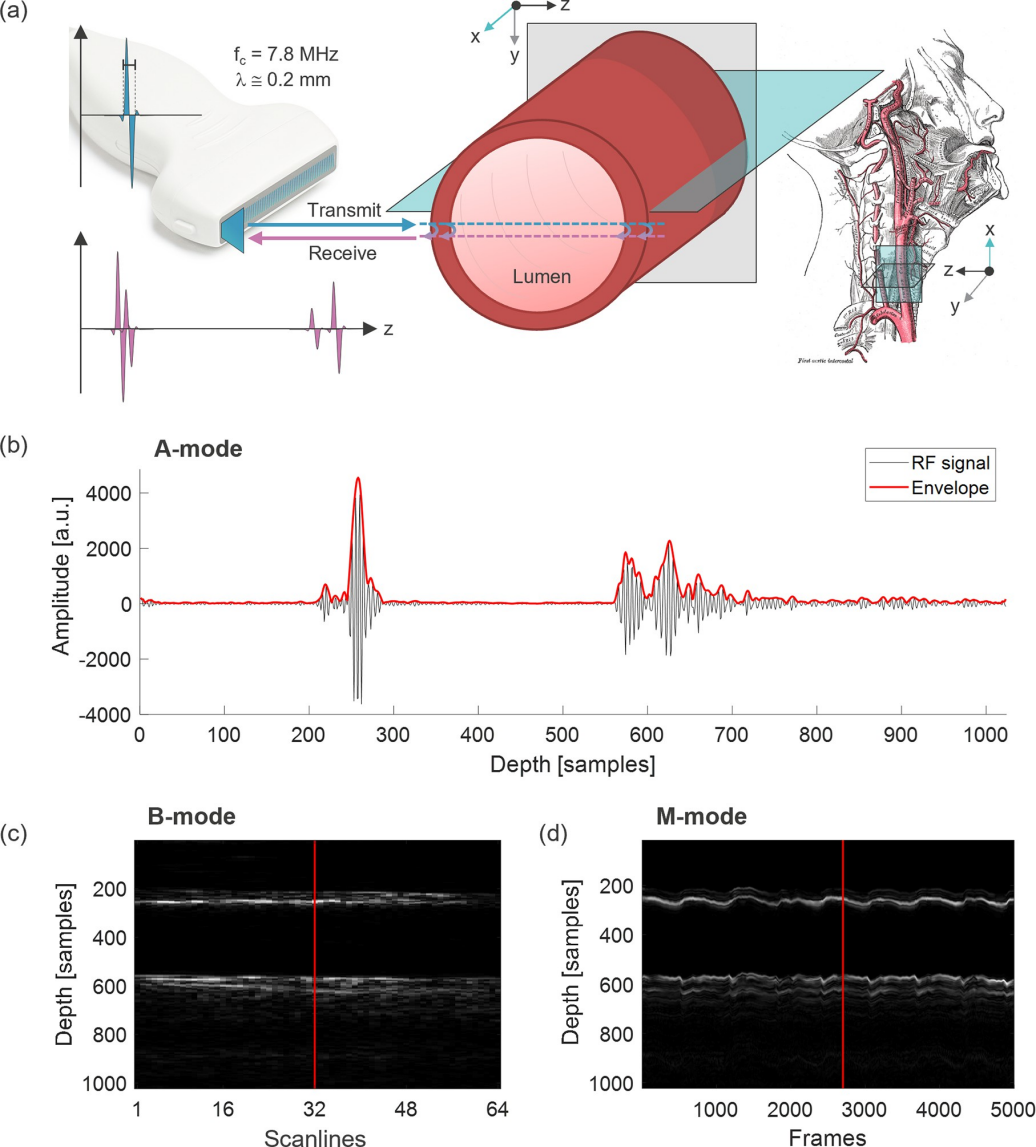
(a) Pulse-echo acquisition of carotid artery. (b) A-mode with radio frequency (RF) ultrasound signal from the center scanline. (c) B-mode of cross-sectional artery. (d) M-mode data of center scanline, recorded at 500 frames per second. By convention, the ultrasound data is presented with depth information along z-axis, scaled at 24.65 μm per sample, and lateral scanlines along x-axis, while the elevational y-axis is less relevant here. f_c_: center frequency, λ: wavelength.

Before revisiting relevant work, it is vital to understand the three basic modes of displaying ultrasound data: 1) A-mode, or amplitude mode, reveals the pulse-echo amplitude information for a single ultrasound transducer element or scanline in depth/axial (z) direction; 2) B-mode, or brightness mode, reveals a 2D spatial contrast image in axial (z) and lateral (x) dimensions along a linear array of transducer elements, and 3) M-mode, or motion mode, reveals the information of a single A-mode scanline over time. Fig 1 illustrates the pulse-echo acquisition with a linear array transducer from a cross-sectional carotid artery, including respective display modes. Note that the radio frequency (RF) signal envelope smoothly outlining the signal magnitude, constitutes the main contrast mechanism based on the ultrasonic echo intensity.

Most prevalent research has tried to address the challenge of (semi-)automatic carotid artery tracking with 2D image processing on B-mode [8,14–16,19], or M-mode [13,20] frames. A less common approach (as it is more challenging to build robust models with less and more noisy data), yet more promising for miniaturized automated solutions (less voluminous data and processing hardware for imaging), is to regress the artery wall positions using the 1-D signal from A-mode frames [9–12,17] from 1 or few adjacent scanlines only. Image processing algorithms using B-mode or M-mode 2-D images are generally computationally more intensive and have higher latency than processing unidimensional A-mode frames. Moreover, B-mode requires an array transducer with multiple scanlines while algorithms using A-mode 1-D signals, in compromise, can be lower in robustness and accuracy.

Effective DSP-based algorithms require clever hand-crafted heuristics [12,17] and labor-intensive manual annotation [21], both of which render personalization challenging. A less explored but very promising alternative are machine learning based algorithms where a model is learned through training from real data in a supervised [22] or unsupervised way [23]. The challenge in this approach is the acquisition of sufficiently large datasets for model training: the larger the model the more data it needs for training to produce a robust and accurate algorithm. For example, to train the models of the algorithms in a previous supervised approach, the authors claim they used data captured from 107 subjects, with 300–2000 A-mode frames (data points) per subject [22]. Datasets of this size are not readily available in general.

Finally, between entirely machine-learning algorithms and DSP-based algorithms there are "hybrid" or ML-assisted approaches where parts of an algorithm pipeline use a trained model from data and the rest relies on heuristics and DSP [15,20,24–27]. This in fact is the more commonplace use of ML, that reflects an incremental adoption in an otherwise DSP dominated field; or some more complex needs that are not easily addressed at the DSP level such as feature extraction and fusion [27].

### Aim of this study

As a step out towards addressing these technology limitations, this paper proposes a machine learning based algorithm pipeline that can *automate* detection and tracking of the artery diameter, without a specialist intervention for annotations and thus self-compensating for the physiological/anatomical variability of different subjects. The proposed pipeline consists of a small set of neural networks each trained on user data that operate in tandem to segment, localize, track, and estimate the artery diameter from one ultrasound scanline. The use of ML algorithms and more recently neural networks in medical ultrasound imaging is not new [28–33], but has been so far confined to non-temporal tasks of image registration, segmentation, and classification of various conditions based on ultrasound images. By contrast to other ML-based offline use-cases, here we take advantage of time-series regression in order to benefit from low computation, low latency, inference on A-mode images, that can be done in a real time streaming application context. For validation, the results are compared against a reference / ground truth those from the DSP-based approach in [17], providing insights on the trade-off between a ML and a DSP-based solution.

## Methods

The proposed solution is a neural-network based processing pipeline that consists of an ROI-Detection module, a tracking module, and a post-processing smoothing module. Each neural network is trained with a small dataset created for this purpose from recordings in a clinical setting. As a starting point and baseline for comparison for the work in this paper, we used a DSP-based approach as reference [17]. In the following sections we outline the data acquisition setup and dataset creation process, and the design of the individual neural networks. For the experiments in this paper the DSP-based reference was developed in Matlab 2018 (Mathworks, Inc.) while the neural-network-based approach was trained and evaluated in Python using the PyTorch framework (https://pytorch.org/). For the hyperparameter tuning and neural architecture search of the neural network models we used grid-search with the help of an Nvidia Quadro M4000 GPU.

### Data acquisition and dataset preparation

We built a dataset based on data acquired from 6 apparently healthy human subjects (4 male, 2 female, 37 ± 7 years of age; mean arterial pressure 86.3 ± 7.6 mmHg), ranging from optimal blood pressure to hypertension, though without diagnosed cardiovascular disease. All subjects gave their written informed consent. The study was reviewed by the institutional review board of the Máxima MC Hospital (Eindhoven, NL) and procedures followed clinical practice guidelines wherever applicable in compliance with the declaration of Helsinki [34]. Data was collected in three repeated measurement sessions, spread over three weeks, to account for physiological intra-subject variability as well as technical measurement bias, e.g., due to sensor reattachment and probe repositioning. All data was recorded at constant room temperature (22°C), in supine position to eliminate the hydrostatic BP component, and with an initial resting phase of 10 minutes to bring hemodynamics and vasomotor tone as close as possible to baseline [35]. Each session, in turn, comprised three interventions. First, 2 minutes in resting condition were recorded for best inter-subject comparability. Second, the subject was asked to perform 2 minutes of paced breathing to induce cyclic BP variation at 7.5 cycles per minute, guided by an acoustic reference signal. Third, to induce a short-term gradual BP and hence diameter increase, the subject was asked to perform a hand grip dynamometer exercise with the sensor free hand for 1 minute at maximal voluntary contraction, followed by 1 minute of recovery. Continuous non-invasive and intermittent blood pressure (BP) readings were obtained using the Finapres NOVA (Finapres Medical Systems B.V., NL). Overall, this setup provides sufficient variability in terms of number of subjects and physiological conditions as well as length of recordings to produce a substantial number of A-mode or B-mode frames (each recording contains 65000–70000 time frames, with 1020 samples in depth on 32 transducer elements) for a general-purpose dataset which also allows exploration of personalization aspects.

**Fig 2:**
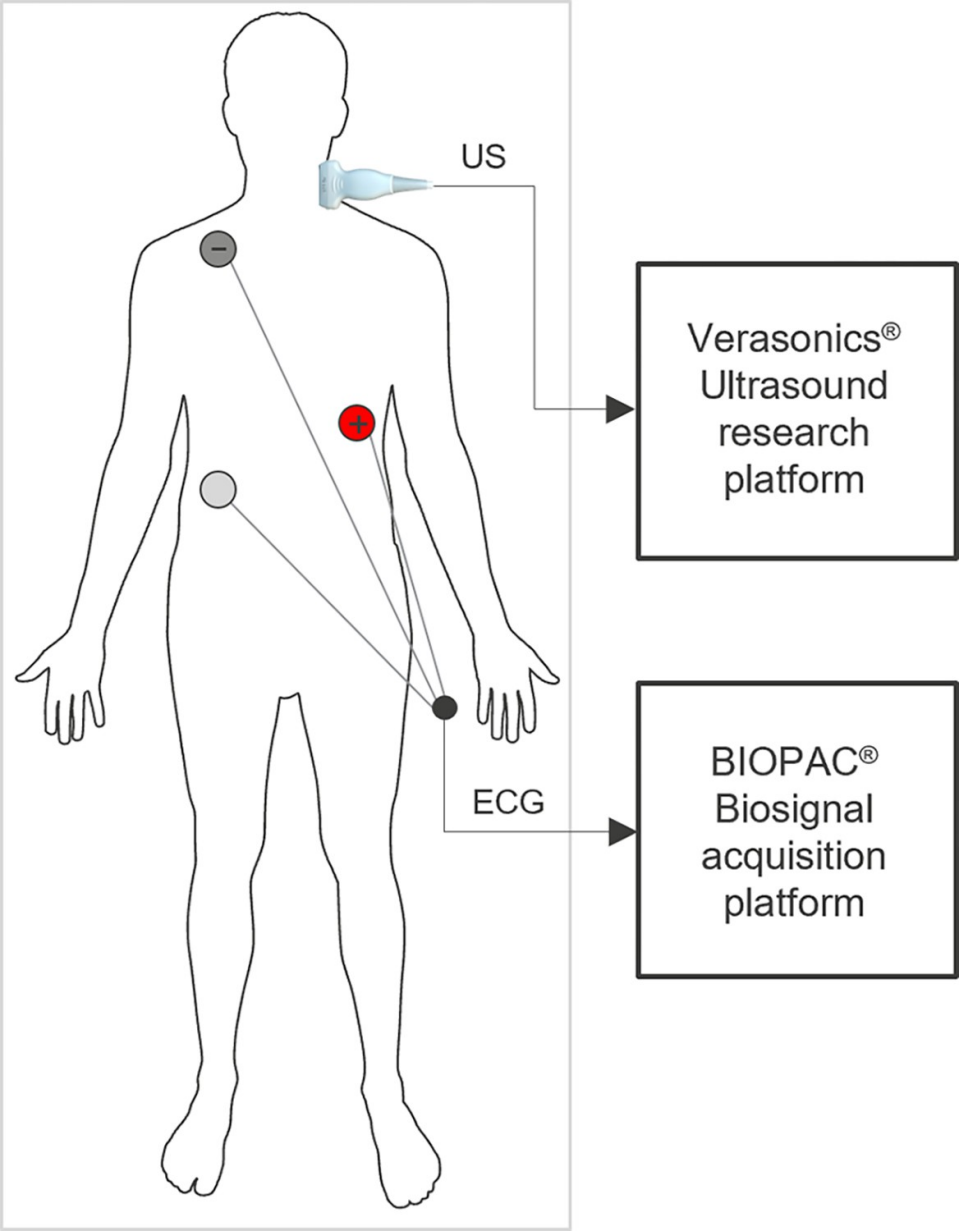
Measurement setup with instrumentation and subject lying in supine position. Ultrasound (US) was acquired with a Verasonics Vantage 64 system (Verasonics Inc., USA) and a transducer (L11-5v) of 128 elements, placed perpendicular to the common carotid artery of the test subjects. The center frequency (fc) of the transducer is 7.8MHz, and the sampling frequency is 500Hz. Simultaneously, an electrocardiogram (ECG) was acquired using a Biopac MP-160 base module (fs = 500 Hz) and ECG100C module (Biopac Inc., USA). Recordings of both modules were synchronized by an external trigger signal.

The measurements setup is shown in Fig 2. The ultrasound transducer deployed in the data acquisition is the L11-5v, and the ultrasound system is a Verasonics Vantage 64 model (Verasonics Inc., USA). The transducer consists of 128 elements in a linear array, working on a center frequency (fc) of 7.8 MHz, acquiring a 19.2 mm wide segment with a custom plane wave sequence of sampling frequency 500 Hz from the center 64 transducer elements. Furthermore, with the center frequency of the RF ultrasound signal and the assumed propagation speed of ultrasound (1540 m/s in soft tissue, 1580–1630 m/s in the arterial wall and 1570 m/s in blood), the wavelength λ is approximately 0.2 mm for a single pulse and hence, the spatial resolution (λ/2, 0.1 mm) satisfies requirements of artery wall detection in the axial resolution. With RF data sampling at 31.25 MHz, a spatial sampling distance of 50 μm can fully recover the original signal with sufficient accuracy. Simultaneously, an electrocardiogram (ECG) was acquired in lead II configuration using a Biopac MP-160 base module (fs = 500 Hz) and ECG100C module (Biopac Inc., USA). Verasonics and Biopac recordings were synchronized by an external trigger signal. The transducer is placed horizontally perpendicular to the common carotid artery of the test subjects, resulting in the maximum detection response in the radial direction.

As illustrated in Fig 1, the acquired ultrasound data are typically organized along 3 dimensions: tissue depth, scanlines (width) and frames (time). The depth dimension corresponds to the half-distance travelled by a single ultrasonic pulse between the transmission and detection of the back-reflected pulse wave. The amplitude in the A-mode representation as well as the brightness in the M-mode representation corresponds to the intensity of the received ultrasound echoes. Along the time dimension, recorded reflections of ultrasound pulses emitted with a pulse repetition frequency of 500 Hz (2 ms per sample) can reveal the temporal evolution of the artery, i.e., pulsatile distension and respiratory movement. The scanlines (width) dimension refers to the linear arrangement of the elements in the transducer as they detect the information on the transverse plane of the artery, generating spatial B-mode images. Without loss of generality, for the machine learning pipeline described in the following sections we only used a single (center) scanline from the transducer, and we process a single pulse-echo signal at a time. In other words, we perform time-series regression on the M-mode plane by processing sequentially A-mode frames.

Ground truth label data was generated from a conventional DSP-based approach [17], which implies an echo tracking algorithm that is commonly used in clinical ultrasound practice [9,23]. This reference dataset was reviewed by experts in vascular ultrasound and, in the rare case of misdetections from the DSP-based algorithm, corrected by manual annotations. Key labels from this data are the arterial lumen center position, the arterial wall positions, and the resulting diameter waveforms in previously specified temporal (2 ms per sample) and spatial (24.65 μm per sample) resolutions.

### Region of Interest (ROI) detection

**Fig 3:**
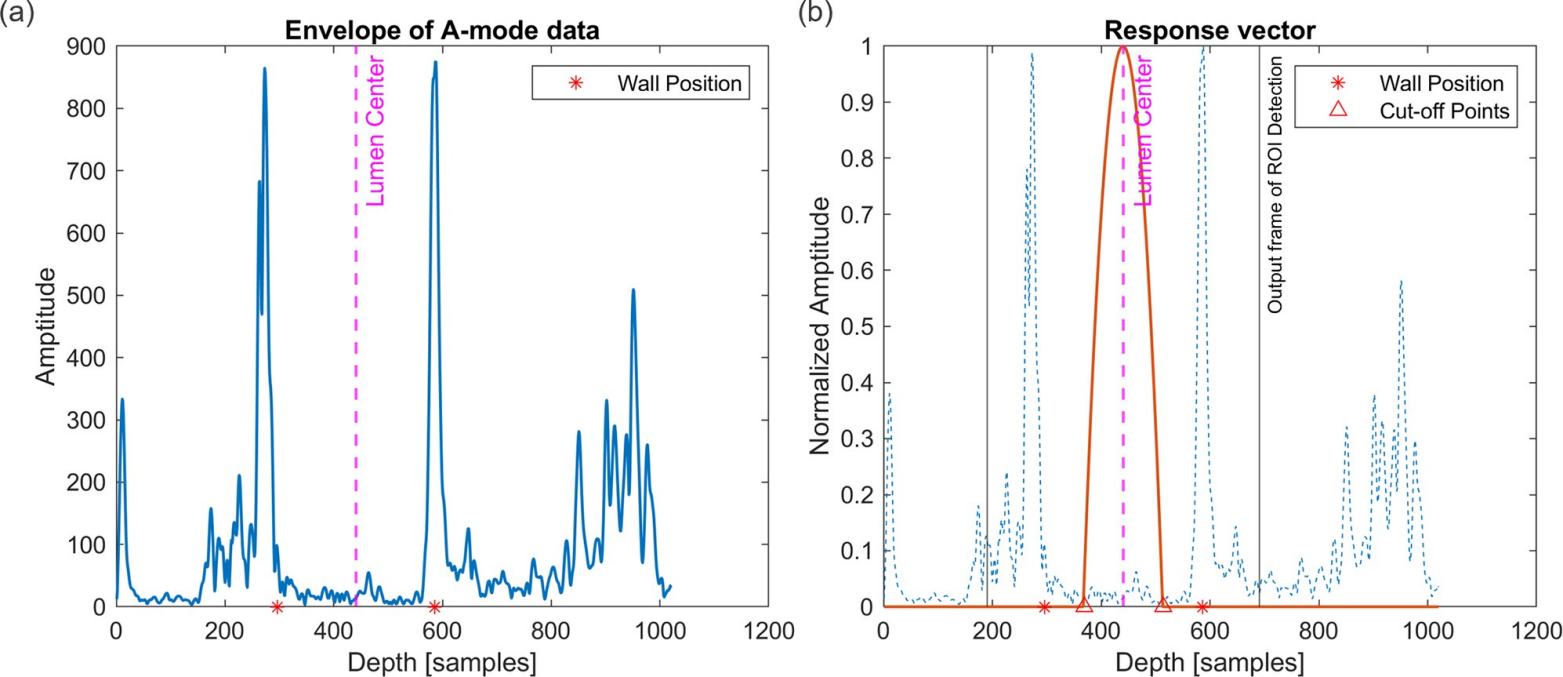
(a) Left plot shows the complete A-mode ultrasound return and the identification of the lumen center as the center of the hypoechoic region. (b) Right plot shows the respective response vector computed as a label for training the ROI detector neural network, as well as the ROI part cropped out for further downstream processing after the ROI detector has been trained. Depth axis sample points can be multiplied by 24.65μm to obtain the actual depth.

The raw ultrasound data is rather noisy due to tissue consistency and measurement artifacts as illustrated in Fig 3, showing an A-mode plot (echo amplitude per axial depth for a single scanline and time instance per sampling period), and an M-mode plot (single scanline echo amplitude in brightness per time instance). While irrelevant low noise may originate from scatterers like blood cells, tissue structures different from the actual arterial wall (e.g. veins, muscles or ligaments) can lead to high-contrast artifacts. To increase the robustness of artery diameter tracking system against these noise artifacts, we decided to introduce a “hard-attention” mechanism that narrows down the field-of-view of the artery tracker to a confined region-of-interest (ROI) within the entire A-mode signal that contains the lumen and arterial walls.

We design a ROI detector model as a first-stage neural network and to train it we exploit the heuristic that the lumen of the artery is a hypoechogenic area whose center is the most quiescent part of the A-mode signal reflections. The input to the network is the envelope (of the signal magnitude) of the A-mode ultrasound echo signal, extracted through a Hilbert transform. As the envelope is a smooth outline of the raw signal magnitude, this also gives us a means to adjust the temporal resolution of the input signal (depth resolution of A-mode) for the sake of confining the neural network’s input layer size for computational efficiency. The output of the network is the location of the lumen center where the ROI window should be centered to clip the A-mode signal. We use a feed-forward convolutional model that tries to regress for every (depth) position the centered distance from the lumen center (for the placement of the ROI window). The reason for training a regressive model with distances instead of as binary classification for each position is to avoid working with largely imbalanced training label data (since in every echo signal series there is only one correct position for the lumen center). The generated labels are therefore error gradient-based masks (we call them “response vectors”) that localize the lumen center for every A-mode ultrasound echo, and are extracted from the ground truth DSP-based approach, following an annotation procedure that will be described in detail later.

**Fig 4:**
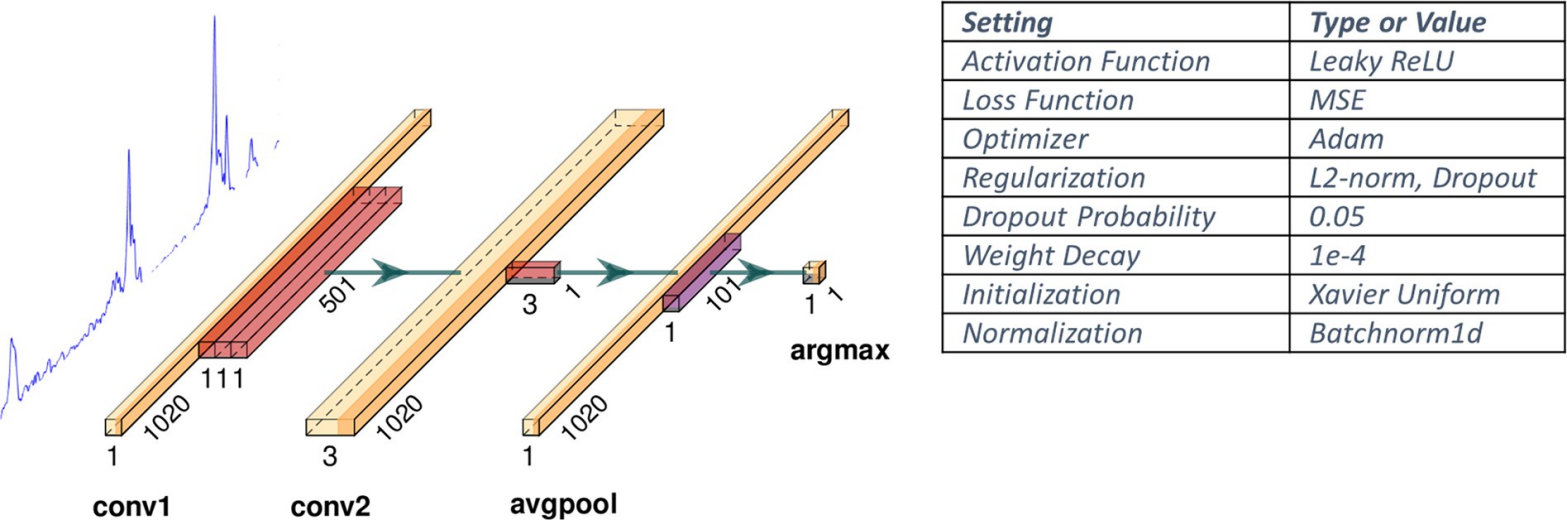
ROI Detection Neural Network Structure and configuration parameters.

The neural network architecture consists of just two 1D convolutional layers followed by an average pooling layer, as shown in Fig 4. The 1D kernel sizes of the first layer relates to the maximum possible anticipated diameter of the artery and should therefore be “wide” enough to account for the demographic physiological variability across different subjects. The number of kernels (output channels) of the input layer can be used to account for spatial variability between the maximum and minimum diameter of the lumen as the artery expands, but since it is merely used to reinforce the depth of the lumen center, only a small number of channels suffices (we used 3); thereby confining significantly the number of required parameters to be trained as well as the number of computations during inference. At the second convolutional layer a small 1x1 kernel summarizes the per channel (distance from lumen center position) information at each depth position, with depth-wise convolution. The two convolutional layers are followed by a parameterless (non-weighted) average pooling layer that consolidates (smoothens) the distance estimates to the lumen center by using information across a neighborhood of depths. Finally, a simple argmax operation produces the estimated position of the lumen center which is used to center and apply a (fixed-size) clipping window over the A-mode signal, the size of which currently matches the input layer kernel sizes. Notably, none of the layers reduce the input width (with appropriate zero-padding employed), because as we see later, we care to preserve the association between layer size and depth information.

For the training of the ROI detector, we generate “response vectors” as labels from the training data, characterizing the lumen region and lumen center. Essentially, the response vector serves as a weight profile for computing an error gradient that measures the degree of misalignment of the center of the ROI window from the lumen center. It is generated based on visual inspection and manual annotation of the approximate position of lumen center in a A-mode or M-mode ultrasound frames. Precision noise in the approximate annotations is easily averaged out during model training. It is essentially this manual intervention by a human specialist, which we wish to factor out by means of the neural network during inference. The lumen center position is identified as the middle point of the hypoechogenic area of the A-mode signal and is defined as

$$P_{C}=(P_{A}+P_{P})/2$$

where *P*_*A*_ is the position of anterior artery wall, *P*_*P*_ is the position of posterior wall.

We set two *cut-off* points halfway between the lumen center and the posterior and anterior walls at the depths where the signal peaks laterally from the lumen center.


$$P_{cut-off(A)}=(P_{C}+P_{A})/2$$

$$P_{cut-off(P)}=(P_{C}+P_{P})/2$$

Within the cut-off region, the Euclidean distance *d*_*i*_ of the current *i*-th depth point *p*_*i*_ towards the lumen center can be computed as

$$d_{i}={\left(p_{i}-P_{C}\right)}^{2},P_{cut-off(A)}<i<P_{cut-off(P)}$$

We finally normalize the distance values in the range (0–1] and use them to set the response vector *R*_*i*_ values as

$$R_{i}=\left\{ \begin{array}{c} 1-\frac{d_{i}}{{\left(P_{C}-P_{cut-off}\right)}^{2}},P_{cut-off(A)}<i<P_{cut-off(P)}\\ 0,Other \end{array}\right.$$

Fig 3 illustrates a response vector centered at the lumen center of an A-mode frame and the respective output of the trained ROI detector model.

Note that since the average pooling layer has been chosen instead of a fully connected layer, for being parameterless (fewer parameters allow model training with less training data without risk of overfitting), we can already measure and correct the misalignment by computing the total pointwise mean square error (MSE) between the response vector and the output of the last convolution layer, then directly applying updates to the two convolution layers’ parameters; instead of backpropagating the error from the output of the average pooling layer. This empirically seemed to be giving a “crisper” gradient for training faster, and with less computations involved.

Finally, to avoid degenerate training and overfitting because of relatively limited range in the depth position of the lumen center across subjects, we have augmented the dataset with translations of the A-frame at different depths. This allows the network to learn to place the ROI at different depths by looking for the signal peaks of the wall positions. The most critical parameterization aspects of the neural network model and its training, that contributed in the results presented later, are given in the table in Fig 4. The choice of the hyperparameters for the regularizer, optimizer, dropout and minimum adequate number of input layer filters were determined empirically based on grid-search. The training in most cases took about 10 epochs after which the error plateaus or improves very slowly (for the specific choice of hyperparameters). Finally, the choice of the network architecture is mainly based on the heuristics described earlier in this section and motivated by the need for compactness.

### Artery distension tracking

Given a ROI of the A-mode frame envelope that contains the artery walls without other high amplitude artifacts, a second stage neural network model is trained to calculate as reliably as possible a running estimate (trace) of the arterial wall distension.

Since the task is one of a temporal regression nature, the apparent candidate neural network model types could include recurrent neural network models, which like low-pass infinite impulse response filters digest the input signal frame-after-frame to produce a smooth distension estimate. However, since the data is univariate and compressible in fast-time, and that predictions are made after an entire A-mode frame is processed, each A-mode frame is possible to be treated as a single data point for feed-forward models as well, which produce diameter estimates for each frame separately and independently from previous estimates. In the latter case smoothing is often required as a post-processing step to eliminate potentially high-frequency artifacts. After experimenting with both, it turns out that between the two options, the latter attains easier good performance, with less training time and data.

**Fig 5:**
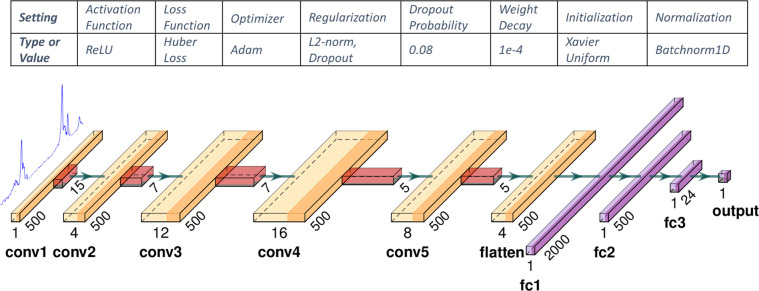
Artery diameter tracking Neural Network Structure and configuration parameters.

After a grid-search exploration and different configurations (numbers of layers, number of channels, sizes of filter kernels), the leanest structure and topology that we resorted to is illustrated in Fig 5. It consists of five convolutional layers of similar width, but incrementally increasing in number of channels and then down-sampling them, followed by a hierarchy of 3 fully connected layers that gradually combine and summarize the convolutional features to compute an estimator of the distension. Between convolutional layers there is batch-normalization. While we up-sample and then down-sample across channels we do not apply max-pooling across layers, and apply zero-padding at the convolution layers, because we care about the relative position information of the arterial walls to estimate the distension, so distorting this information with max-pooling makes training harder and deteriorates the performance.

We trained this model with the Huber loss, instead of the more common MSE loss, because of its robustness in face of the abrupt high-amplitude difference variations of the A-mode signal across depths as the ultrasound pulse enters and exits the lumen region of the artery. As with the ROI the key parameterization and training configuration that contributed to the results presented later are provided in the table in Fig 5. The training hyperparameters were determined empirically based on grid-search. We needed about 20 training epochs for this configuration for the error to reach an initial plateau. The architecture of the network was inferred (as discussed above) partly heuristically and partly empirically by starting from a two-layer model progressively increasing depth and channel width to reach an acceptable performance.

### Post-processing / filtering

Finally, as alluded above, the output of the feed-forward neural network estimator for the artery diameter is not a smooth waveform because of the per-single point estimation noise. Therefore, to eliminate the high-frequency noise, we postprocess the output trace with a Savitzky-Golay filter [36], which is commonly applied to time-series signals to reduce high-frequency noise without distorting the signal tendency. The order of the Savitzky-Golay filter is set to 5, and the window size to 31 samples (corresponding to 62 ms), hence preserving actual higher frequency components in the waveform. For comparison we also considered a simple moving average FIR filter with the same window size. As the filter parameters are not trainable, the entire pipeline consists of approximately 1 million trainable parameters in total (combined, the ROI detection and diameter tracking networks).

### Evaluation

The ultrasound dataset consists of recordings from 6 subjects, obtained in repeated sessions under varying physiological conditions. From these recordings, we extract approximately 600.000 data points (i.e. A-mode frames) in total. Following a leave-one-subject-out cross-validation scheme to obtain unbiased results, each subject is iteratively left out and tested on the neural network models for ROI detection and diameter tracking, respectively trained on the approximately 500.000 data points from all other subjects. For the reported results, per neural network model for ROI detection and diameter tracking, the average of the 6 unbiased model performances is reported. Training results are quantified by the respective loss function among the 6 models. Ultimately, to evaluate the NNs performance and sensitivity to changing inputs, correlations and error metrics are computed between their predicted results, (i.e. the detected lumen center and arterial diameter) and corresponding label data from the conventional DSP-based approach described in [17], serving as ground truth reference.

## Results

**Fig 6:**
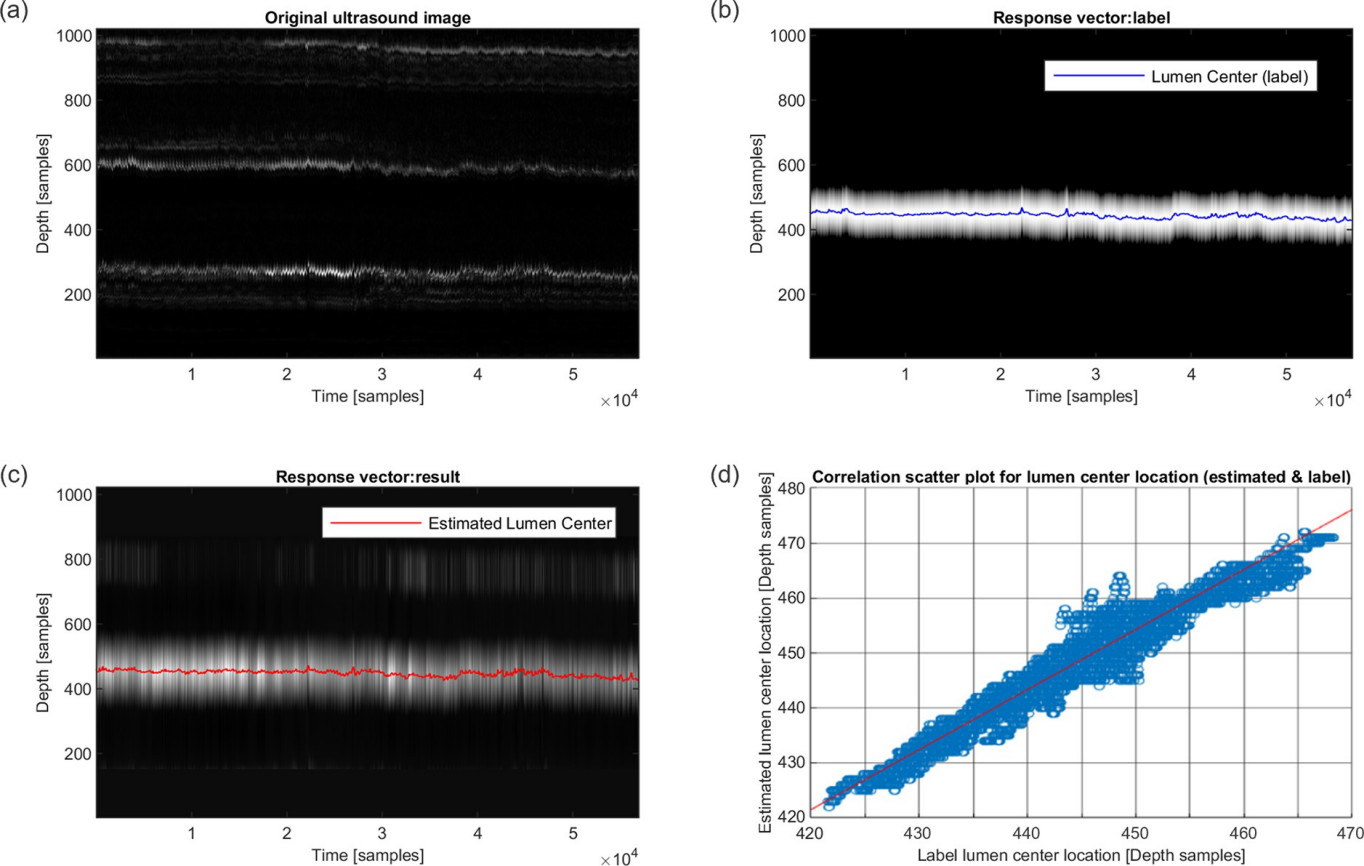
Typical example of M-mode recordings during 2 minutes of rest (a) with labeled lumen center (b) and ROI network results for lumen centers (c) and their correlations (d). Depth axis sample points can be multiplied by 24.65 μm to obtain the actual depth. Conversion factor from time samples to time is 500 samples per second.

The result section is divided into the ROI detector network and the diameter tracking network. Firstly, Fig 6 illustrates the qualitative performance of the ROI detector network by providing a typical example. Fig 6(A) is the M-mode signal that contains the arterial walls near sample depth 300 and 600 as well as other artefacts around sample depth 200, 650, 900 and 1000. Fig 6(B) shows in M-mode only the response vector labels (white region) and the lumen center (blue line). Fig 6(C) shows in M-mode the output of the ROI detector before the argmax operation, depicting as probabilities (intensity) the likely location of the lumen center. Despite the artifacts at locations 100, 800, and between 400–500, the argmax operation will only select the highest intensity point (red line).

To quantitatively validate the performance of the ROI detector, we calculated the correlation between the DSP-based label signal of the lumen center and the estimation, resulting in Pearson correlation *r* = 0.9742, while the coefficient of determination is *R*^2^ = 0.7194, and the concordance correlation coefficient is CCC = 0.8388. Data appears to be normally distributed based on the moments of the distribution of the differences between ground truth and predicted values (mean = 4.3252, median = 4.3897, skewness = -0.14, kurtosis relative to Normal distribution = 0.2315) and the corresponding p-value for lack of a linear relationship (H_0_), based on a 2-sided t-test, is virtually 0 (t-value = 763.7324). Furthermore, Table 1 reports the average per subject Mean Absolute Error (MAE) in the estimation of the lumen center. The overall across all subjects’ average absolute error is 6.4724×24.65 μm≈0.2 mm. For reference, the average carotid artery diameter in the study population is 7.5 mm.

**Table 1:** Average results of the ROI network in samples. Conversion factor from samples to depth and diameter distances is 24.65 μm per sample. MSE: Mean squared error (normalized without dimension), MAE: Mean absolute error.

Data	MSE (Response Vectors)	MAE (Lumen Center) [Samples]
Subject 1	0.00714	4.5126
Subject 2	0.01028	6.8277
Subject 3	0.00862	5.3201
Subject 4	0.01039	6.0514
Subject 5	0.00821	10.1607
Subject 6	0.01196	5.9619
Avg across subjects	0.00943	6.4724

**Fig 7:**
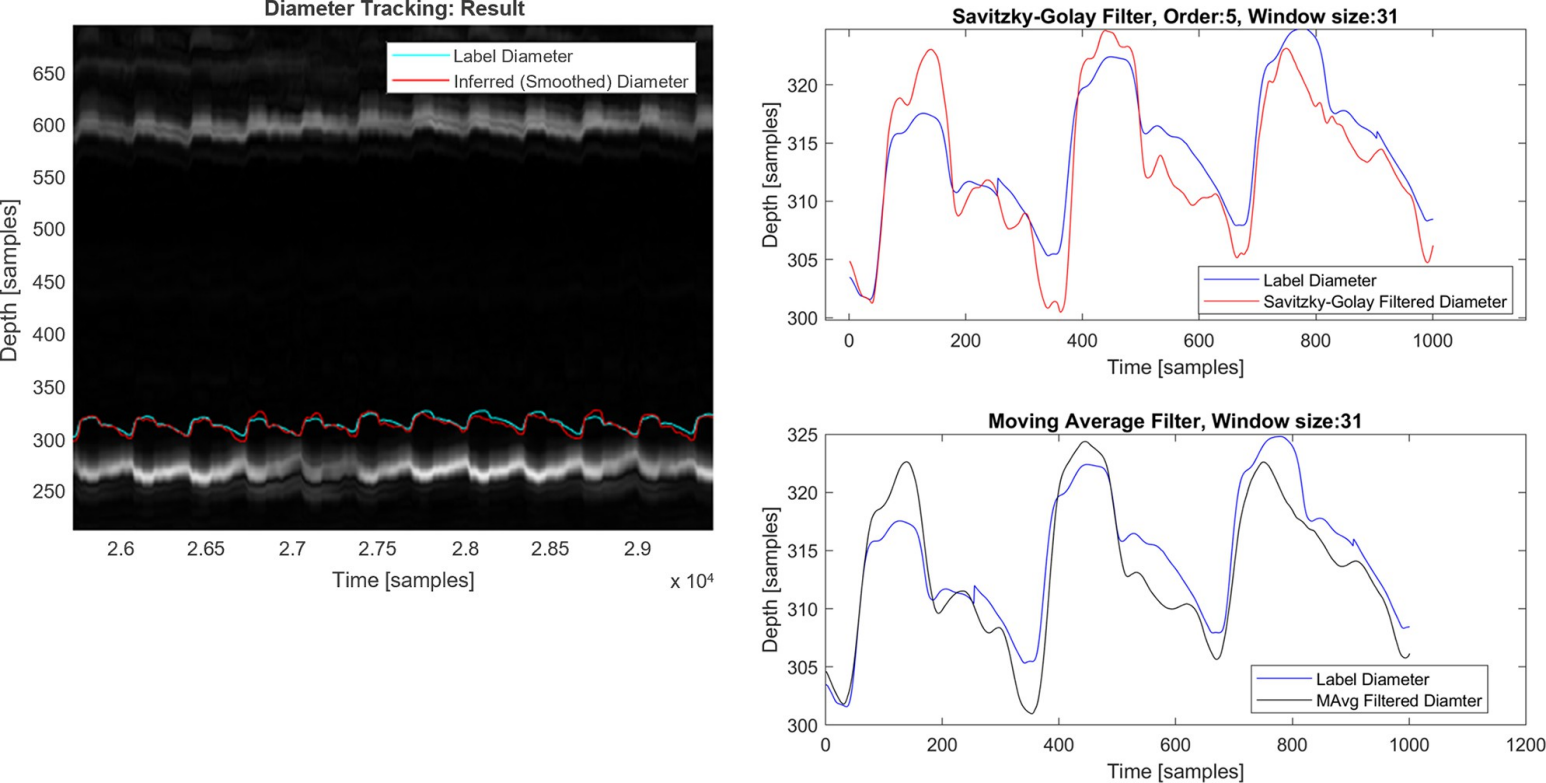
Example of diameter tracking network results over M-mode (left) with reference label diameter and neural network output, respectively smoothed with Savitzky-Golay (top right) and moving average filter (bottom right). Conversion factor from samples to depth and diameter distances is 24.65 μm per sample. Conversion factor from time samples to time is 500 samples per second.

Regarding the output of the diameter tracking network, Fig 7 shows the inferred signals of the artery diameter over time overlayed with the reference. The first (visual) confirmation is that the inferred signal tracks the reference without temporal drifts and tracks the systolic and diastolic variations (upstroke, max slope in the first derivative, etc.) with high fidelity. A closer observation on a zoomed-in region shows that while the temporal alignment is robust, the inferred signal does not exactly match the amplitude and microvariations of the reference and moreover has a superimposed small high-frequency noise component. This noise component was anticipated and is almost eliminated by the smoothing filter while preserving relevant physiological information, e.g., in the region around the systolic foot. On the other hand, the small mismatch between the two signal amplitudes is likely due to imperfections both on the neural network side as well as in the reference, which is derived from a DSP-based pipeline [17].

To quantify the discrepancy in Table 2, we report the average root-mean-square error (RMSE) per subject as well as across all subjects, which amounts to 9.0805×24.65 μm≈0.22 mm. Since RMSE is sensitive to outliers, we also report the median-absolute-deviation (MAD), which is robust to outliers and in this case is 3.1198×24.65 μm≈0.077 mm. Given an average artery diameter of 7.5 mm for the subjects in this study, this corresponds to a ~1% error. Finally, the Pearson correlation coefficient between prediction and reference waveforms averaged across all test subjects is *r* = 0.8692, corresponding to a coefficient of determination *R*^2^ = 0.7243, respectively 72% explained variability.

**Table 2:** Averaged error metrics in samples obtained from the arterial diameter tracking network. Conversion factor from samples to depth and diameter distances is 24.65 μm per sample. MSE: Mean squared error (normalized without dimension), RMSE: Root mean squared error, MAD: Mean absolute deviation.

Data	Huber Loss	Average Diameter (Reference / Label) [Samples]	MSE	RMSE [Samples]	MAD [Samples]
Subject 1	9.6217	292.7785	205.57	14.3377	4.1147
Subject 2	3.3608	286.2240	17.87	4.2507	1.7039
Subject 3	7.8044	311.1213	109.57	10.4715	4.3095
Subject 4	7.0987	319.3344	82.85	9.1027	3.6739
Subject 5	4.0786	302.1867	58.95	7.7113	1.6738
Subject 6	6.0920	314.1656	74.12	8.6092	3.2429
Avg across subjects	6.3387	304.3018	91.49	9.0805	3.1198

## Discussion

This work proposes a system for automated tracking of the artery diameter consisting of a cascade of two neural networks (NNs): the first network implementing a hard-attention model for a ROI detection that isolates the region of the wall positions, feeding into the second network responsible for the diameter tracking. Attention models (soft-attention in discrete language applications and hard-attention in image processing) are becoming commonplace in deep learning literature for dealing with the computational efficiency and/or robustness of very high dimensional inputs. In most cases one seeks to train the attention mechanism end-to-end or in tandem with the downstream image processing model. It involves a suitably engineered cost-function and (fully, partially or self) supervised learning [37–39], or in the more challenging cases reinforcement learning [40–43]. This typically requires a substantial amount of training data to avoid overfitting as the end-to-end model ends up with a very large number of parameters; and, in the case of reinforcement learning, also a considerable amount of time. By contrast, in the work presented, the two models are trained independently. This is because we want the models to be sufficiently shallow for computational economy, and also for being trainable with a small subject cohort to keep training logistics low, i.e., re-training or fine-tuning to account for demographic adaptation, should be possible with a limited number of subjects. Training two smaller independently regularized models is therefore effective with fewer data points, while enforcing the a-priori known conditional dependence between the two networks. Overall, much of the computational economy of the proposed solution comes from design choices that are application domain specific. For example, by contrast to general purposed hard attention mechanisms here we do not try to dynamically extrapolate the size of the attention window, instead we keep it fixed given the limited range variation in the diameter of the artery, and we only track dynamically the lumen for positioning of the ROI window. For the same reason, the number of input convolutional filters (2nd layer channels) does not need to be larger than 3, which turned out to be sufficient after some experimentation. Furthermore, by keeping the size of the ROI detector layers aligned with the input dimensionality that reflects the arterial wall depth resolution, the estimation of the lumen location does not need to be computed but is implicitly represented by the index of the argmax-ed neuron at the output layer.

In the design of the ROI detection model, we used the heuristic that the lumen region of the artery is hypoechoic, in similar spirit as the DSP approach in [17]. While tracking of the lumen center in that work is based on a hand-crafted cross-correlation filter kernel, here we essentially let the neural network model *learn* a couple of analogous correlation kernels in the CNN and combine them in sophisticated ways to account for physiological and measurement variability. An additional advantage from the automated *learning from data* approach is that the resulting models can encode salient features and characteristics that are sometimes missed or not visible to a human modeler. This information may cover trivialities, like the onset of a new cardiac cycle, but also complexities like retrieving the arterial location after gradual drift or incidental interruptions in the images due to motion artifacts, e.g., from coughing or swallowing. Consequently, a prospective system implementation may not need to rely on ECG gating with periodic resets and potential signal discontinuities as described in [17], and thereby facilitate applicability in clinical practice. The ML-based approach is particularly encouraging from a computational efficiency point of view because it does not rely on complex analytical signals. Although it shows noisier waveforms compared to the DSP approach, the finer movements between neighboring A-mode frames can be compensated through the smoothing filter. Moreover, the deliberate choice of a polynomial smoothing filter over a conventional moving average filter has shown not only to preserve but also amplify such salient features around the systolic foot, which may enable advanced pulse waveform analysis [44]. However, their physiological foundation remains to be further validated.

Limitations of the presented work pertain to both physiological and technical considerations. Firstly, the proposed method was developed based on a small-scale cohort. On the one hand, the good NN pipeline performance provides a solid proof of concept within the presented inter-subject (i.e. differences in age, gender, and baseline BP) and intra-subject (i.e. induced BP changes) variability. On the other hand, physiological variability and, hence, inferring the generalizability of the proposed models, is still limited. Therefore, future research will not only cover wider ranges in baseline determinants of healthy subjects (e.g. age, gender, and BP)[45], but also relevant patient cohorts with cardiovascular diseases (e.g. atherosclerosis and carotid stenosis). This will further challenge the proposed method, while expanding training data and affirming its generalizability.

Technical limitations span from the ultrasound modality to processing architectures. The proposed NN architecture is tailored to high-quality ultrasound data. On the one hand, the heuristics-based ROI detection and diameter estimation might be less effective when ultrasound data is collected in lower quality configurations (e.g. resolution, noise level, etc.), which may limit generalization of the models. On the other hand, the processing methods in this work are confined to operate on raw ultrasound data resolution, whereas complex analytical signals with preserved phase information, e.g. used in [17], would allow for sub-sample arterial motion detection. In this sense, this processing pipeline does not yet fully exploit the raw ultrasound data information, although potential gains in fineness of the results should remain balanced with computational complexity.

Future work will advance in both the ultrasound modality and processing refinements. Regarding the ultrasound modality, the proposed neural network for ROI detection in combination with a large field-of-view US transducer may enable autonomous acquisition without the need for manual alignment of the transducer to the artery. The machine-learning (ML) pipeline uses the A-mode signal from a single (center) scanline, even though we used an array-based transducer of 64 scanlines. Utilizing effectively the remaining scanlines is left as future work. Although computational complexity shall be balanced, a straightforward approach would be to leverage all 64 scanlines by means of a ML learning ensemble of models (one for each scanline), which is expected to increase precision and fidelity while reducing tracking variability [46–48]. Eventually, with wider lateral information, methods may be expanded, e.g. for B-mode image segmentation methods to assess the degree of carotid stenosis [49].

Given that the scope of this work has been a feasibility study, aiming to provide proof of concept for ML-based ultrasound signal processing, a systematic quantitative comparison against the reference DSP approach on the computational and power-efficiency aspects is still missing and targeted as future work. Such a comparison necessitates on one hand testing on a small range of embedded micro-controllers or hardware neural accelerators (e.g. AD’s MAX78000 Cortex, GreenWave’s Gap9[50], imec’s SENECA [51]), and on the other hand a number of *accelerator-friendly* refinements for the neural-network models to make them more compact and more resource efficient (quantization, pruning, distillation, etc). The benefit of an ML-based approach is that even such optimizations can be automated through in-training procedures while *learning from data*, and in stark contrast with tedious handcrafting in DSP based workflows.

## Conclusion

This work proposed and evaluated a machine learning neural-network based pipeline to detect and track the carotid artery diameter from an ultrasound stream of A-mode frames. Our evaluation showed that the proposed solution results in only 0.6–1.4% deviation in the tracking of the carotid diameter by comparison to the reference, coming from a DSP-based solution, with *R*^2^ = 0.7243.

To the best of our knowledge, the herein presented work is one of the first successful implementations of machine-learning for an ultrasound time-series regression task, i.e., for temporally tracking the carotid artery diameter. The proposed approach is a fully automated solution that does not require any intervention from a specialist (e.g., for annotations/markers). Ultimately, the reliance only on A-mode frames, renders the solution promising for miniaturization and deployment in on-line clinical and ambulatory monitoring.

The main advantage of a machine learning approach over a DSP approach is the automatic extrapolation of the right tracking function from data, rather than having to improvise heuristics and perform tedious manual fine-tuning. One of the commonly argued disadvantages of a learning-from-data approach is that it requires a lot of data to learn large computationally expensive (deep) models. However, we have shown that by exploiting application domain knowledge, we can arrive at effective models which are neither too large, nor require much data for training, and may also be computationally very affordable and appealing for embedded deployment.
